# The Shiga toxin 2 production level in enterohemorrhagic *Escherichia coli* O157:H7 is correlated with the subtypes of toxin-encoding phage

**DOI:** 10.1038/srep16663

**Published:** 2015-11-16

**Authors:** Yoshitoshi Ogura, Shakhinur Islam Mondal, Md Rakibul Islam, Toshihiro Mako, Kokichi Arisawa, Keisuke Katsura, Tadasuke Ooka, Yasuhiro Gotoh, Kazunori Murase, Makoto Ohnishi, Tetsuya Hayashi

**Affiliations:** 1 Department of Bacteriology, Faculty of Medical Sciences, Kyushu University, 3-1-1 Maidashi, Higashi-ku, Fukuoka 812-8582, Japan; 2 Division of Microbiology, Department of Infectious Diseases, Faculty of Medicine, University of Miyazaki, 5200 Kihara, Kiyotake, Miyazaki 889-1692, Japan; 3 Genetic Engineering and Biotechnology Department, Shahjalal University of Science and Technology, Kumargaon, Sylhet-3114, Bangladesh; 4 Environmental Science Division, Fukuoka City Institute for Hygiene and the Environment, 2-1-34 Jigyouhama, Chuou-ku, Fukuoka 810-0065, Japan; 5 Department of Preventive Medicine, Institute of Health Biosciences, University of Tokushima Graduate School, 3-18-15, Kuramoto, Tokushima 770-8504, Japan; 6 Department of Microbiology, Graduate School of Medical and Dental Sciences, Kagoshima University, 8-35-1 Sakuragaoka, Kagoshima 890-8544, Japan; 7 Department of Bacteriology, National Institute of Infectious Diseases, 1-23-1 Toyama, Shinjuku-ku, Tokyo 162-8640, Japan

## Abstract

Enterohemorrhagic *E. coli* (EHEC) causes diarrhea and hemorrhagic colitis with life-threatening complications, such as hemolytic uremic syndrome. Their major virulence factor is Shiga toxin (Stx), which is encoded by bacteriophages. Of the two types of Stx, the production of Stx2, particularly that of Stx2a (a subtype of Stx2), is a major risk factor for severe EHEC infections, but the Stx2 production level is highly variable between strains. Here, we define four major and two minor subtypes of Stx2a-encoding phages according to their replication proteins. The subtypes are correlated with Stx2a titers produced by the host O157 strains, suggesting a critical role of the phage subtype in determining the Stx2a production level. We further show that one of the two subclades in the clade 8, a proposed hyper-virulent lineage of O157, carries the Stx2 phage subtype that confers the highest Stx2 production to the host strain. The presence of this subclade may explain the proposed high virulence potential of clade 8. These results provide novel insights into the variation in virulence among O157 strains and highlight the role of phage variation in determining the production level of the virulence factors that phages encode.

The evolution of pathogenic bacteria is often promoted by the horizontal gene transfer (HGT) of virulence-associated genetic determinants^1^,^2^. Although most, if not all, HGT events are mediated by mobile genetic elements (MGEs), such as bacteriophages (simply called phages), plasmids and transposons, these MGEs are often regarded as mere vehicles carrying the virulence determinants. However, MGEs themselves evolve rapidly and display remarkable genetic variation, even between closely related elements. A particularly high degree of genetic variation is exhibited by phages, which have shorter generation times and can easily recombine with other phages, as evidenced by the widespread genetic mosaicism observed in phage genomes^3^,^4^,^5^. Such variations among phages may directly or indirectly affect the evolution of the host bacteria to which the phage transduces virulence determinant(s); however, the biological significance of genetic variation among phages has not yet been fully elucidated. Although infection and lysogenization by toxin-encoding phages converts otherwise non-pathogenic bacteria into highly virulent pathogens^6^,^7^,^8^,^9^,^10^, the production levels of such toxins often vary among bacterial strains^11^,^12^,^13^,^14^. Variations in toxin-encoding phages may be linked to such differences in toxin production levels, but this possibility has not yet been fully explored in any pathogenic bacteria.

Shiga toxin (Stx) is the key virulence factor of enterohemorrhagic *Escherichia coli* (EHEC), which causes diarrhea and hemorrhagic colitis with life-threatening complications, such as hemolytic uremic syndrome (HUS), although EHEC possesses a broad range of virulence factors, many of which are needed for the full virulent phenotype^15^. Stx, a member of the AB-type toxin family, inhibits protein synthesis in host cells by depurinating a specific adenine residue of the 28S rRNA^15^. Stxs are classified as Stx1 and Stx2, both of which contain several subtypes^16^. EHEC strains produce one or more Stx subtypes^15^. Strains of serotype O157 (EHEC O157), the most prevalent EHEC worldwide, produce Stx1, Stx2a and Stx2c, alone or in combination. The level of Stx2c production by EHEC O157 is generally thought to be very low^17^,^18^,^19^. Importantly, Stx2-producing strains cause more severe infections than Stx1-producing strains^20^. In fact, purified Stx2 is 1,000 times more toxic to human renal endothelial cells than Stx1^21^.

The *stx* genes are encoded by lambdoid phages and are located downstream of the phage late gene promoter^22^. Although *stx1* is under the control of the iron-regulated authentic promoter, the expression of *stx2* depends strongly on the late promoter^23^,^24^,^25^,^26^,^27^,^28^. When Stx2-encoding phages (Stx2 phages hereafter) are integrated into the host chromosome (lysogenic state), *stx2* is repressed by the phage CI repressor. However, once the SOS response is provoked in the host cells treated with DNA-damaging agents, such as mitomycin C (MMC), the activated RecA protein stimulates the autocleavage of the CI repressor, resulting in phage induction and replication. Upon induction, early genes are first expressed, after which the late promoter-dependent expression of late phage genes is induced by the action of the antiterminator protein Q. Protein Q is produced during early gene expression and subsequently acts on the transcription terminator, which is located immediately downstream of the late promoter. Therefore, Stx2 production is tightly coupled with phage induction and replication, which suggests that variations among Stx2 phages could affect the Stx2 production level of each EHEC. In fact, the level of Stx2 production varies remarkably even between EHEC strains of the same serotype (i.e., EHEC O157)^11^,^12^,^17^,^18^,^19^,^29^,^30^. Considerable genomic diversity has also been found among phages encoding the same Stx2 subtype (i.e., Stx2a phages)^19^,^31^,^32^.

Here, we present the results of a comprehensive analysis of 123 Stx2-positive O157 strains and their Stx2 phages. Our data indicate that Stx2c phages are highly homogeneous. However, most Stx2a phages are classified into four major subtypes according to their replication proteins, and these subtypes are correlated with the Stx2a production levels by host strains. We further show that EHEC O157 strains belonging to clade 8, which has been proposed to be a highly pathogenic clade of O157^19^,^33^,^34^,^35^, can be separated into two subclades by a high-resolution phylogenetic analysis. One of these two subclades carries the subtype of the Stx2a phage that confers the highest Stx2a production to the host strain. The presence of this subclade may explain the proposed high virulence potential of clade 8.

## Results

### Phylogenetic clades, *stx2* subtypes and Stx2 phage integration sites of 123 O157 strains

The 123 *stx2*-positive O157 strains analyzed in this study were all human strains isolated in various regions of Japan between the 1990s and 2006 (Supplementary Table S1), with the exception of EDL933, which was isolated in the United States in 1982^36^. The strain set included 15 strains from asymptomatic carriers. A phylogenetic clade analysis was conducted via a rapid differentiation method based on the sequences of eight single nucleotide polymorphism (SNP) sites^35^,^37^. This analysis revealed that 4, 11, 20, 2, 6, 64 and 12 strains belonged to clades 1, 2, 3, 4/5, 6, 7 and 8, respectively, and 4 strains were untypeable (Table 1). A restriction fragment-length polymorphism (RFLP) analysis of the *stx2* genes^38^ showed that 49 and 59 of the strains contained either *stx2a* or *stx2c* alone, respectively, whereas the remaining 15 strains contained both *stx2a* and *stx2c* (Table 1).

The chromosomal integration sites of the Stx2 phages in each strain were determined using a long PCR-based method (see Methods). All the Stx2 phages were found to be integrated into one of three known integration sites: 39 Stx2a phages were integrated into the *wrbA* locus, 25 Stx2a phages were integrated into the *argW* locus, and all 74 of the Stx2c phages were integrated into the *sbcB* locus (Table 1). Intriguingly, most of the strains carrying an Stx2a phage in *wrbA* belonged to clades 1, 2 and 3 (35/39) and were *stx1*-positive (36/39) (Table 1). In contrast, all 12 strains belonging to clade 8 carried an Stx2a phage in the *argW* locus, whereas four of them carried an Stx2c phage as well. None of the assessed clade 8 strains were *stx1*-positive. The majority of the strains in the other clades (clades 4 to 7) carried the Stx2c phage alone in the *sbcB* locus (59/72) or together with the Stx2a phage in *argW* (11/72). Thus, the distribution of the Stx2 phages appears to be clade dependent.

### Stx2 production levels

**Figure 1:**
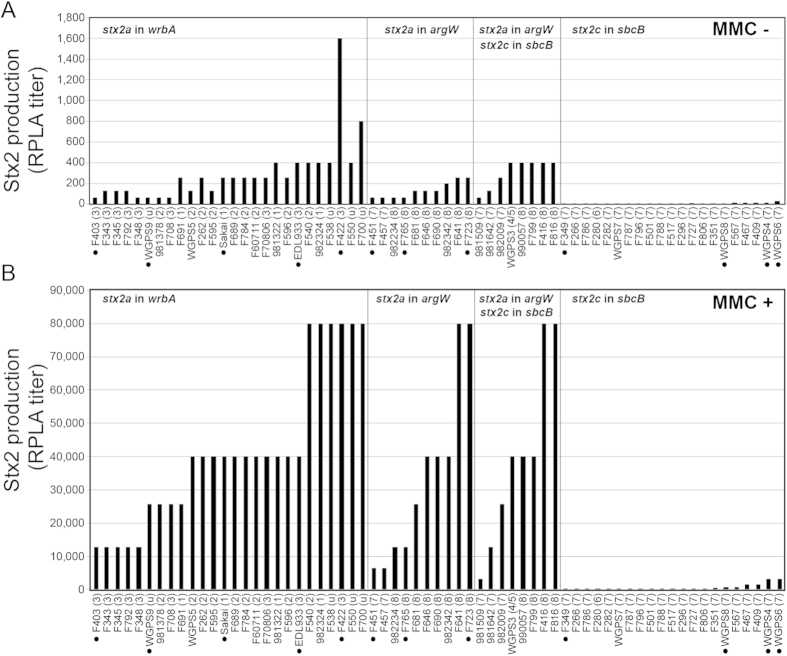
Highly variable Stx2 production levels among O157 strains. The Stx2 production levels of the 65 O157 strains are shown. The supernatants of the polymixin B-treated cultures were prepared from the 65 O157 strains without or with the MMC treatment (panels (**A**,**B**), respectively). The Stx2 concentration in each supernatant was measured using the VTEC-Reversed Passive Latex Agglutination (VTEC-RPLA) assay kit, and the resulting RPLA titers are shown. The Stx2 prophages of the strains indicated by the dots were sequenced in this study. The strains were categorized according to their stx2 subtypes and Stx2 phage integration sites.

From the 123 strains, we selected 65 that represented various combinations of Stx2 subtypes, Stx2 phage integration sites and phylogenetic clades, and we measured their Stx2 production levels in the presence and absence of MMC by means of a reversed passive latex agglutination (RPLA) assay. Even in the absence of MMC, Stx2 production was detected in all the strains. A remarkable strain-to-strain variation in toxin titer (which ranged from 2 to 1,600 in RPLA titer) was also observed (Fig. 1 and Supplementary Table S1). Consistent with previous reports^17^,^18^,^19^, MMC treatment did not strongly enhance Stx2 production in the strains carrying *stx2c* alone. In contrast, Stx2 production was remarkably enhanced by MMC in almost all the strains carrying *stx2a,* and the observed Stx2 production levels were highly variable among the strains. The levels of MMC-induced Stx2 production by these strains were significantly correlated with those in the absence of MMC (Supplementary Fig. S1), but there was no significant relation between Stx2 production and the clade of the strain or the integration site of the Stx2a phage. Recently, Neupane *et al.* showed that clade 8 strains overexpressed *stx2* in both RNA and protein levels compared to clade 1–3 strains^11^. In this study, however, only four clade 8 strains were analyzed and one of them produced a very low level of Stx2. Thus, although the authors concluded that the increased *stx2* expression is characteristics of clade8 strains, it is not exclusive to this clade and not all strains within clade 8 overexpress *stx2*. The Stx2 production patterns of *stx2a*/*2c* double-positive strains were similar to those of *stx2a* single-positive strains. This is consistent with a previous study by Kawano *et al.*^18^, and suggests that the Stx2 production levels of the *stx2a*/*2c* double-positive strains are largely representative of the levels of Stx2a production by each strain.

### Genomic diversity of Stx2 phages in O157

**Figure 2:**
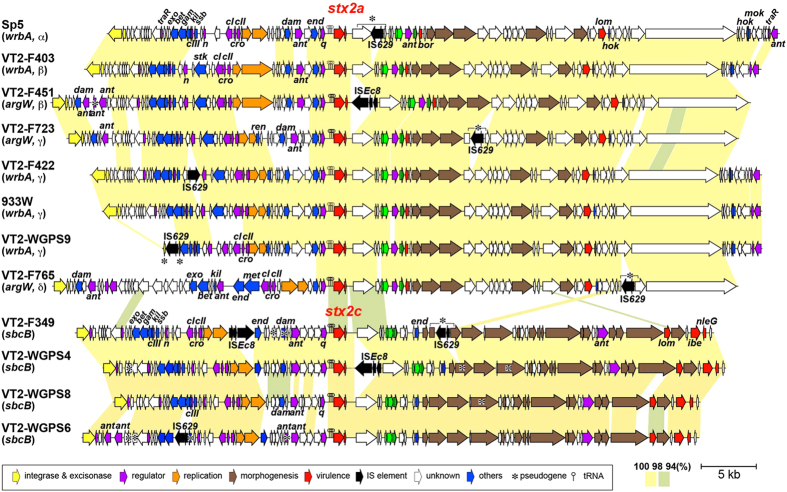
Genome comparison of Stx2 phages. The genome structures of the six Stx2a and four Stx2c phages sequenced in this study are drawn to scale. Two previously sequenced Stx2a phages of O157 strains Sakai and EDL933 (Sp5 and 933W, respectively) are also included. Homologous regions are indicated by shading, and the sequence identities are indicated by different colors. The VT2-WGPS9 phage contains a large deletion encompassing the sequence from int to bet; this deletion was likely generated by an IS-mediated event. Integration sites of each phage and subtypes of Stx2a phages (α, β, γ and δ; see the main text and Fig. 3 for more details) are also indicated.

We selected 10 O157 strains that contained only one Stx2 phage (Table 2) and determined the genome sequences of their Stx2 prophages using a fosmid-based method. These strains produced different levels of Stx2, and their Stx phages carried either *stx2a* or *stx2c* and were integrated into *wrbA, argW* or *sbcB* (three strains had the Stx2a phage in *wrbA*, three had the Stx2a phage in *argW*, and four had the Stx2c phage in *sbcB)*. A dot-plot analysis of the genome sequences of these 10 phages and of Sp5 (the Stx2a phage of the O157 strain Sakai^39^, also known as VT2-Sakai) and 933W (the Stx2a phage of the O157 strain EDL933^40^) revealed that the Stx2a phages exhibited overall sequence similarities to each other, irrespective of their integration sites (*wrbA* or *argW*), whereas the Stx2c phages were highly divergent from the Stx2a phages (Supplementary Fig. S2). A more detailed comparison of the Stx2a phages revealed a notable degree of intra-group genomic diversity in their early regions (upstream of *stx2*) but only minor structural variations in their late regions (Fig. 2). As previously suggested^33^,^41^, the Stx2c phages of the O157 strains were highly homogeneous. The genomic structures of the four Stx2c phages sequenced in this study were well conserved, with only a few small replacements and strain-specific insertion sequence (IS) insertions being detected (Fig. 2).

### Subtyping of the Stx2a phages of O157 and comparison of the Stx2 production levels among the strains carrying each subtype of the Stx2a phage

**Figure 3:**
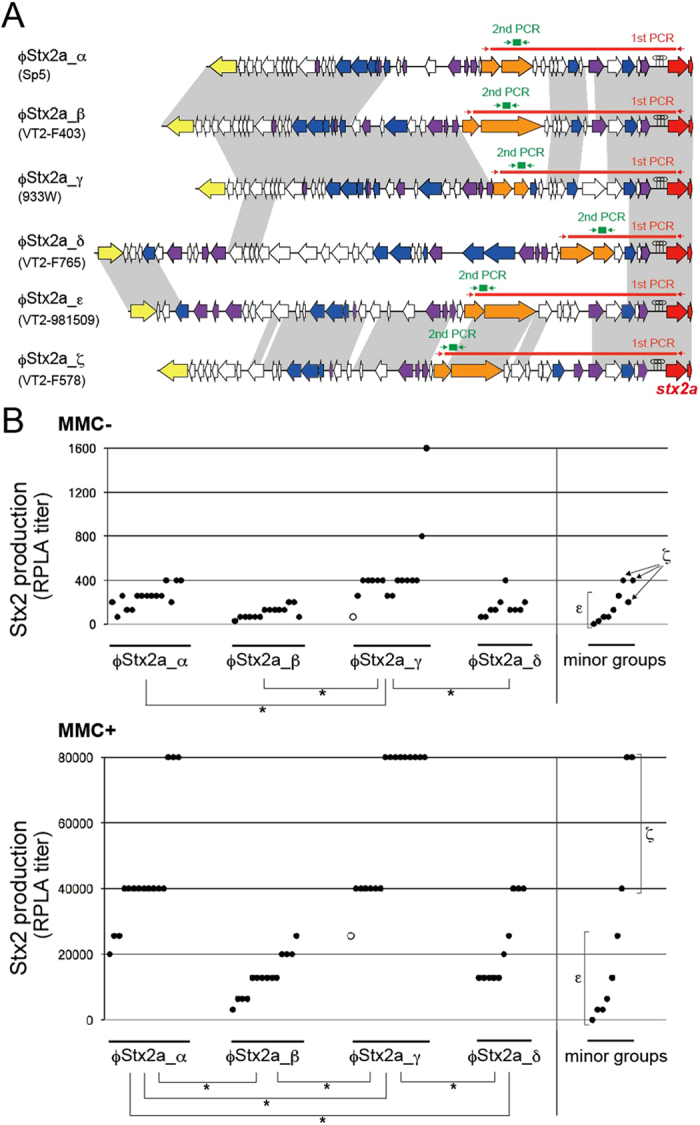
Stx2a phage subtypes and the comparison of Stx2 production levels among the strain groups carrying each subtype of the Stx2a phage. (**A**) The six Stx2a phage subtypes that were identified and a two-step PCR-based subtyping system are shown here. Based on structural variations in the phage early region, the Stx2a phages were classified into six subtypes (α, β, γ, δ, ε and ζ). Representative genomic structures of each subtype are shown. The locations of the primers used in the first and second PCRs to subtype the Stx2a phages are also indicated. (**B**) The Stx2 production levels (RPLA titers) of the 54 stx2a-positive O157 strains with or without MMC treatment (shown in the lower and upper panels, respectively) were determined and compared between the six strain groups carrying each subtype of the Stx2a phage. The experiments were repeated at least three times per strain, and representative data are shown. The statistical significance was calculated using an ANOVA followed by Tukey’s multiple-comparisons procedure (*P < 0.05). Note that, among the strains containing the ϕStx2a_γ phage, a strain indicated by open circle corresponds to strain WGPS9, the Stx2a phage of which could not be excised from the host chromosome due to the deletion of its int and xis genes.

Genomic variations in the early region of the Stx2a phages (Fig. 2) may be linked to the variation in Stx2 production levels among the *stx2a*-positive strains (Fig. 1). In this regard, the variation of replication proteins between the Stx2a phages is particularly interesting; we identified four types of replication proteins among the eight Stx2a phages examined (Fig. 2). We therefore grouped these phages into four subtypes, ϕStx2a_α (Sp5), ϕStx2a_β (VT2-F403 and VT2-F451), ϕStx2a_γ (933W, VT2-F422, VT2-F723 and VT-WGPS9) and ϕStx2a_δ (VT2-F765), according to the type of replication proteins. The replication genes completely differed in sequence between the four subtypes (Supplementary Fig. S3). We therefore developed a two-step PCR-based subtyping system for the Stx2a phages using an *stx2a*-specific primer (stx2-R) and primers specific to the replication genes of each subtype (Fig. 3A; primer sequences are available in Supplementary Table S2). Using this system, the Stx2a phages from 55 of the 64 *stx2a*-positive strains (including the *stx2a*/*2c* double-positive strains) were categorized into one of the four subtypes (15 ϕStx2a_α, 14 ϕStx2a_β, 16 ϕStx2a_γ or 10 ϕStx2a_δ); however, the 9 remaining *stx2a*-positive strains were untypeable (Supplementary Table S1). We therefore determined the sequences of early regions of all the untypeable Stx2a phages. From the comparison of the sequences, these untypeable phages were categorized into two groups (ϕStx2a_ε and ϕStx2a_ζ) (Supplementary Fig. S4 and Table S1). These two groups possessed similar replication genes. But they were very different from those of the four major subtypes and the 5′-half of the O gene homologs completely differed between the two subtypes (Supplementary Fig. S3). Therefore, two-step PCR systems to subtype these minor groups were also constructed using the unique sequences in their replication genes (Fig. 3A).

We then compared the Stx2 production levels of the *stx2a*-positive strains and found significant differences in the Stx2 production levels among the four major groups of strains carrying each subtype of the Stx2a phage (Fig. 3B). In both the presence and absence of MMC, the ϕStx2a_γ group produced more Stx2 than the other groups (note that a ϕStx2a_γ subtype phage indicated by an open circle could not be excised from the chromosome due to the deletion of its *int* and *xis* genes). The ϕStx2a_α group also produced more Stx2 than the other two groups (ϕStx2a_β and ϕStx2a_δ) in the presence of MMC, although no significant differences were observed in the absence of MMC. This result suggests that strains carrying phage ϕStx2a_γ or ϕStx2a_α (particularly the former) can produce higher Stx2 levels than strains carrying other Stx2a phage subtypes. Among the strains carrying two minor subtypes of Stx2a phage, those carrying ϕStx2a_ζ may also produce a higher level of Stx2. However, more strains need to be examined to perform a reliable statistic analysis.

### High-resolution phylogenetic analysis and Stx2a phage subtype distribution in O157 clade 8 strains

**Figure 4:**
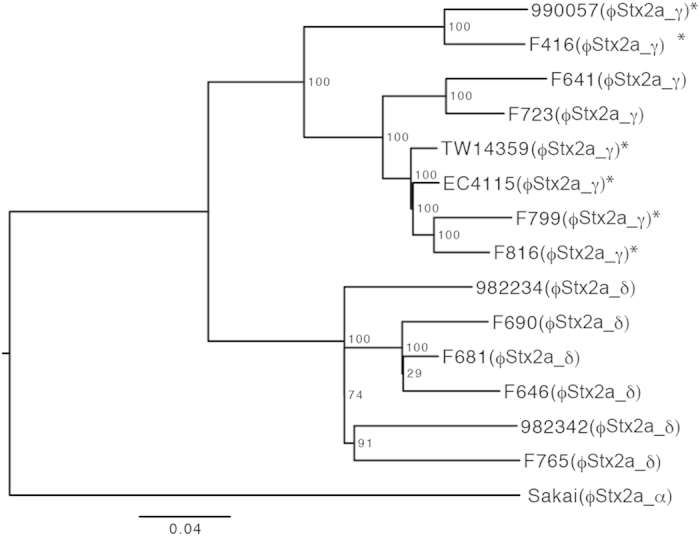
High-resolution phylogenetic analysis of the O157 clade 8 strains. Based on 975 SNP sites on the chromosomal backbone, which were identified using a genome sequence comparison among the K-12 MG1655, O157 Sakai and 14 O157 clade 8 strains, a maximum likelihood phylogenetic tree was generated in RAxML ver. 7.2.8 using the GTR-GAMMA model. The reliabilities of the tree’s internal branches were assessed using bootstrapping with 1,000 pseudoreplicates. The scale bar represents the number of substitutions per site. Strain TW14359 (a completely sequenced clade 8 strain isolated from the spinach-associated outbreak in the USA in 2006) was used as a reference. Strain EC4115 (another completely sequenced clade 8 strain isolated from the spinach-associated outbreak) and strain Sakai (clade 3) were also included in this analysis. The other 12 strains were sequenced in this study. The subtypes of the Stx2a phage in each strain are indicated in parentheses. Asterisks indicate the strains carrying an Stx2c phage in addition to the Stx2a phage.

Clade 8 has been proposed to be a highly pathogenic clade of O157^34^,^35^. Although the mechanism(s) underlying the high pathogenicity of the strains in clade 8 has not yet been well elucidated, these strains may produce a higher level of Stx2^11^. However, whereas 6 of the 12 clade 8 strains examined in this study were found to carry ϕStx2a_γ, the remaining six strains carried ϕStx2a_δ (Supplementary Table S1). To determine the phylogenetic relationship between these two types of clade 8 strains, we performed a whole genome sequence-based, high-resolution phylogenetic analysis of the clade 8 strains using high-quality draft genome sequences obtained from the Illumina MiSeq sequencer (Fig. 4 and Supplementary Table S3). We included three previously sequenced O157 strains in this analysis as a control: two clade 8 strains (TW14359 and EC4115, both isolated in the “2006 spinach outbreak” in the United States^19^,^33^) and one clade 3 strain (Sakai). Our results revealed that the clade 8 strains were divided into two distinct subclades (referred to as subclades 8a and 8b). Importantly, the subclade 8a strains, which included TW14359 and EC4115, carried ϕStx2a_γ exclusively, and all the subclade 8b strains carried ϕStx2a_δ (Fig. 4). Furthermore, only subclade 8a strains (six of the eight strains, indicated by asterisks in Fig. 4) contained Stx2c phages in addition to Stx2a phages. It should also be noted that, in both subclades, the Stx2a phages were integrated into the same locus (*argW*); however, it is currently unknown whether the two subclades acquired their different Stx2a phage subtypes independently or whether the exchange of phage subtypes occurred in either or both subclade(s) after their separation. It is also unknown whether (or to what extent) the difference in genetic background between the two sublineages affects the Stx2a production level and potential virulence of the strains in each sublineage.

## Discussion

As confirmed in this study (Fig. 1), the Stx2 production levels of the O157 strains were highly variable. However, the genetic determinants that specify these production levels have long remained unknown. Because Stx2 is a major risk factor for severe EHEC infections, including those caused by *E. coli* O157^20^,^21^, the factors responsible for high Stx2 production are of great interest. Through a comprehensive analysis of 123 *stx2*-positive O157 strains and their Stx2 phages, including the genome sequencing and comparison of 12 representative Stx2 prophages, we showed that the Stx2a phage subtype is a critical factor that determines the Stx2 production level in O157.

Based on the genome sequences of the six Stx2a phages determined in this study and two previously determined, we defined four major Stx2a subtypes, each having distinct replication proteins. The complete genome sequences of additional Stx2a phages, such as VT2phi_272 (HQ424692), Min27 (EU311208), Xuzhou21 (CP001925), SS17 (CP008805) and the Stx2a prophages from the “spinach outbreak” strains TW14359 and EC4115, are available in public databases^19^,^33^,^42^,^43^,^44^. However, these phages were either very similar or nearly identical to at least one of the six Stx2a phages analyzed in this study (Supplementary Fig. S5). In fact, most Stx2a phages of the strains tested could be categorized as one of these four major Stx2a phage subtypes using the PCR-based subtyping system developed in this study (Supplementary Table S1). Although sequence analysis of the nine untypeable phages further identified two minor Stx2a phage subtypes (Supplementary Fig. S4), it is likely that most of the major subtypes of Stx2a phages of the O157 strains have been identified. In addition, we showed that the Stx2c phages of O157, whose genome structures were distinct from those of the Stx2a phages, were highly homogeneous (Fig. 2).

Our data from the Stx2a phage subtyping and the determination of the Stx2 production levels of the O157 strains indicated a significant correlation between the Stx2a phage subtype and the Stx2 production level by O157. Other factors, such as genetic backgrounds associated with the lineages of host strains, could also affect the Stx2 production level by O157, but the Stx2a phage subtype may be one of the critical factors to determine it and the Stx2 production levels of each O157 strain appear to be predictable by subtyping their Stx2a phages. Of the four major subtypes, ϕStx2a_γ conferred the ability to produce the highest level of Stx2 in the host strains, and strains carrying ϕStx2a_α produced more Stx2 than strains carrying the other two subtypes of Stx2 phages.

The efficiency of phage induction is thought to be critical in determining the level of *stx2* expression, which depends on the phage late gene promoter^27^,^28^,^45^. However, variations of the *cI* repressor genes, which encode a master regulator of phage induction, and the *q* genes, which encode an antiterminator that allows the *stx2* gene expression from the phage late promoter, cannot account for these differences (Supplementary Fig. S6). The early regions of ϕStx2a_β and ϕStx2a_γ phages exhibited the greatest similarity among the four subtypes; these subtypes differed only in the types of replication genes, the presence of genes encoding Ren (a phage exclusion protein) and several hypothetical proteins (Figs 2 and 3A). However, strains carrying ϕStx2a_β and ϕStx2a_γ exhibited remarkable differences in Stx2 production (Fig. 3B). These divergent genes (or gene sets) are thus candidate factors that determine the efficiency of phage induction and therefore the level of Stx2 production.

Regarding the relationship between phage subtype and Stx2 production levels, the homogeneity of the Stx2c phages may also be important; this homogeneity could be linked to the low level of *stx2c* gene expression that is a general feature of this Stx2 subtype (Fig. 1). The relationship between Stx2 phage type and Stx2 production levels in non-O157 EHEC strains is also an issue of medical and scientific importance that requires additional research, particularly in the case of EHEC serotypes O26, O111, O103, O121, O145 and O45, all of which are considered to pose a particularly high risk^46^,^47^.

Another important finding of this study is the identification of a sublineage of clade 8 (named subclade 8a), which has acquired ϕStx2a_γ. It appears likely that the presence of this subclade could, at least partly, account for the proposed high virulence potential of clade 8^19^,^33^,^34^. A large-scale statistical analysis of the relationship between the subclade (i.e., the Stx2a phage subtype) and the symptoms and disease severities of patients infected by clade 8 strains is required to verify this hypothesis. However, strains TW14359 and EC4115, which were isolated in the “2006 spinach outbreak” that resulted in remarkably high rates of hospitalization (51%) and HUS (16%)^19^,^33^,^34^, were both included in subclade 8a (Fig. 4). Our preliminary analysis of single nucleotide polymorphisms (SNPs) specific to each subclade indicated that subclade 8b corresponds to the previously described 87–14 lineage in Cluster 1^48^,^49^, and the major branch of subclade 8a corresponds to the TW14359 lineage in the same cluster, whereas the minor branch comprised of strains 990057 and F416 exhibited a chimeric feature of the 87–14 and TW14359 lineages in terms of SNP composition.

Finally, it should also be mentioned that ϕStx2a_γ is also distributed in clade 3 and other clades (Supplementary Table S1). Further high-resolution phylogenetic analyses, combined with Stx2a phage subtyping and measurements of Stx2 production levels, may reveal additional highly pathogenic lineages of O157. In such lineages, the potential role of Stx1 and its bacteriophage in toxin expression or the attenuation of virulence may also be re-evaluated^50^,^51^.

In conclusion, we defined that the Stx2a phage subtypes of EHEC O157, which may be a critical factor to determine the Stx2 production level. Differences in phage induction efficiency among the subtypes, which appear to be specified by the types of replication proteins, may account for the differences in Stx2 production levels among the host strains. The proposed high virulence potential of clade 8 could be explained, at least partly, by the presence of subclade 8a, which has acquired ϕStx2a_γ, a phage that can confer the highest Stx2 production levels to the host strains. Thus, our data contribute to a better understanding of the virulence potential of O157 and provide novel insights into the medical importance of bacteriophage variation, which is closely linked to the production levels of the virulence factors encoded by these phages.

## Materials and Methods

### Bacterial strains, culture conditions and isolation of the genomic DNA

Of the 123 O157 strains examined (Supplementary Table S1), the Sakai and EDL933 strains were isolated from large outbreaks that occurred in 1996 and 1982 in Japan and the United States, respectively^36^,^52^. The remaining 121 strains were human isolates collected from various regions of Japan between 1990 and 2006. These strains included seven strains (WGPS3 to WGPS9) that we previously analyzed using the Whole Genome PCR Scanning method^53^. The strain set also contained 16 strains from asymptomatic carriers, which were isolated from food handlers and workers in day-care centers or some other occupations that are obligated to undergo periodic stool examinations. All the strains were routinely grown on Lysogeny Broth (LB) agar or in LB medium at 37 °C. The genomic DNA was purified from 2 ml of an overnight culture of each strain using the Genomic-tip 100/G and Genomic DNA buffer set (QIAGEN, Valencia, CA) according to the manufacturer’s instructions.

### Detection and subtyping of *stx* and phage integration site determination

The detection of the *stx1* and *stx2* genes via PCR and the subtyping of the *stx2* gene via a RFLP analysis have been previously described^38^,^54^. The integration sites of the Stx2 phages were determined using long PCR with the stx2-R primer and a combination of primers specific to each flanking region of the four known Stx2 phage integration sites (the *wrbA*, *argW*, *sbcB* and *yehV* loci). Long PCR was performed using the LA Taq PCR kit (Takara Shuzo, Kyoto, Japan), with 1 ng of genomic DNA used as the template for a two-step amplification program involving 30 cycles of 20 sec at 98 °C and 16 min at 69 °C (or at 66 °C if unsuccessful at 69 °C). All the primers used in this study are listed in Supplementary Table S2.

### Determination of the Stx2 production level

The bacterial cells were inoculated into 2 ml of CAYE medium (Denka Seiken Co., Ltd., Tokyo, Japan) and were grown to the mid-log phase at 37 °C with shaking. MMC was added to the culture (to a final concentration of 500 ng/ml). After 3 h of incubation, polymixin B (Denka Seiken; final concentration 10,000 U/ml) was added, and the incubation continued for 1 h at 37 °C with mixing every 10 min. The culture supernatants were then obtained by centrifugation (7,700 ×*g* for 10 min at 37 °C) and were used for the assay. The MMC-untreated samples were similarly prepared but without the addition of MMC. The supernatants were serially diluted in 96-well V-bottom microtiter plates using the dilution buffer contained in the VTEC-Reversed Passive Latex Agglutination (VTEC-RPLA) assay kit (Denka Seiken). The Stx2 titer of each sample was determined using the VTEC-RPLA assay kit according to the manufacturer’s instructions.

### Sequencing of the Stx2 prophage genomes

Fosmid libraries were constructed using pCC1 (Epicentre, Madison, WI, USA) as previously described^55^. From each fosmid library, *stx2*-containing clones were screened using PCR with *stx2A*-specific primers. Of the *stx2*-positive clones, two clones that encompassed the first and second halves of the Stx2 prophage genome, respectively, were selected for each of the examined strains; these clones were sequenced using shotgun sequencing followed by primer walking. The sequences thus obtained were annotated using the Microbial Genome Annotation Pipeline (MiGAP)^56^ and were manually curated using IMC-GE software (In Silico Biology, Inc., Kanagawa, Japan). The genome sequences of the 10 Stx2 phages sequenced in this study have been deposited in the DDBJ/EMBL/GenBank databases (accession numbers; AP012529–AP012540).

### Subtyping of the Stx2a phages

The Stx2a phages were subtyped using two-step PCR (Fig. 3A). The first PCR was performed using primers targeting the *stx2A* gene (stx2-R) and the replication proteins specific to each subtype. The same PCR condition as that used for long PCR to detect phage integration sits was employed. The second PCR was performed using the first PCR product (10,000 times diluted) as a template and KAPATaq polymerase (Kapa Biosystems, Woburn, MA, USA) with 30 cycles of 20 sec at 98 °C, 30 sec at 58 °C and 1 min at 72 °C; the primers for this step targeted the genes for the replication proteins specific to each subtype. Sequences of all primers used for Stx2a phage subtyping are shown in Supplementary Table S2.

### High-resolution phylogenetic analysis of clade 8 strains

To perform genome sequencing on the Illumina MiSeq sequencing platform (Illumina, San Diego, CA, USA), genomic DNA libraries were prepared from each of the 12 clade 8 strains using the Nextera DNA Sample Prep kit (Illumina). The pooled libraries were subjected to multiplexed paired-end sequencing (2 rounds of 151 cycles). The sequence reads were assembled using the Velvet version 1.2.05 software^57^. Contig sequences from each clade 8 strain and the chromosome sequences of K-12 MG1655^58^, O157 Sakai^52^ and O157 EC4115^33^ were aligned with that of O157 TW14359^19^ using MUMmer version 3.22^59^ to identify the conserved backbone of these strains and its SNP sites. We found that a 2,976,129-bp sequence of the TW14359 chromosome (its total length) was conserved among all the strains examined, with >98% sequence identity and >1,000-bp alignment length. To exclude any SNPs in recombinogenic regions from the analysis, SNP clusters (>2 SNPs within 100 bp) were removed. Finally, RAxML ver. 7.2.8^60^ was used to construct a maximum likelihood phylogenetic tree inferred from the concatenated alignment of 975 SNP sites located on the conserved backbone (the GTR-GAMMA model of nucleotide substitution, 10 replicate runs and 1,000 bootstraps).

### Statistical analyses

Statistical significance was calculated using an analysis of variance (ANOVA) followed by Tukey’s multiple-comparisons procedure (Fig. 3B). Statistical correlations were analyzed using Pearson’s correlation coefficient (r) (Supplementary Fig. S1).

## Additional Information

**How to cite this article**: Ogura, Y. *et al.* The Shiga toxin 2 production level in enterohemorrhagic *Escherichia coli* O157:H7 is correlated with the subtypes of toxin-encoding phage. *Sci. Rep.* **5**, 16663; doi: 10.1038/srep16663 (2015).

## Supplementary Material

Supplementary Information

## Figures and Tables

**Table 1:** Phylogenetic clades and stx2 subtypes of strains used in this study.

*Stx2* subtype	Integration site	No. of strains*									
		Clade 1	Clade 2	Clade 3	Clade 4/5	Clade 6	Clade 7	Clade 8	Clade 9	Untypeable	Total
*stx2a*	*wrbA*	4 (4)	11 (7)	20 (19)	0	0	0	0	0	4 (4)	39 (34)
	*argW*	0	0	0	0	0	2 (0)	8 (0)	0	0	10 (0)
*stx2c*	*sbcB*	0	0	0	1 (0)	2 (0)	56 (23)	0	0	0	59 (23)
*stx2a* + *stx2c*	*argW & sbcB*	0	0	0	1 (1)	4 (0)	6 (0)	4 (0)	0	0	15 (1)
total		4 (4)	11 (7)	20 (19)	2 (1)	6 (0)	64 (23)	12 (0)	0	4 (4)	123 (60)

^*^The number of strains that have the *stx1* gene is indicated in parenthesis.

**Table 2:** Stx2 phages analyzed in this study.

Phage	Strain	*stx2* subtype	Stx2a phage subtyping	Integration site	Length	No. of CDSs	Stx2 RPLA titer		Reference
							MMC−	MMC+	
Sp5	Sakai	*stx2a*	φStx2a_a	*wrbA*	62,708	93	256	40000	Makino *et al.*, 1999.
VT2-F403	F403	*stx2a*	φStx2a_b	*wrbA*	63,369	87	64	12800	This study
VT2-F422	F422	*stx2a*	φStx2a_γ	*wrbA*	63,252	91	1600	80000	This study
933W	EDL933	*stx2a*	φStx2a_γ	*wrbA*	61,670	85	400	40000	Plunkett *et al.*, 1999.
VT2-WGPS9	WGPS9	*stx2a*	φStx2a_γ	*wrbA*	56,224	74	64	25600	This study
VT2-F451	F451	*stx2a*	φStx2a_β	*argW*	64,900	86	64	6400	This study
VT2-F723	F723	*stx2a*	φStx2a_γ	*argW*	62,274	88	256	80000	This study
VT2-F765	F765	*stx2a*	φStx2a_d	*argW*	63,552	78	64	12800	This study
VT2-WGPS4	WGPS4	*stx2c*	—	*sbcB*	58,538	85	16	3200	This study
VT2-WGPS6	WGPS6	*stx2c*	—	*sbcB*	57,849	82	32	3200	This study
VT2-WGPS8	WGPS8	*stx2c*	—	*sbcB*	55,023	80	4	800	This study
VT2-F349	F349	*stx2c*	—	*sbcB*	59,872	88	2	2	This study
