# Supplementary material for: The Shiga toxin 2 production level in enterohemorrhagic Escherichia coli O157:H7 is correlated with the subtypes of toxin-encoding phage
Source: Sci Rep. 2015 Nov 16;5:16663. doi: 10.1038/srep16663 (PMC4645166; doi:10.1038/srep16663)
Supplement: Supplementary Information [file srep16663-s1.pdf]

Supplementary data

The Shiga toxin 2 production level in enterohemorrhagic *Escherichia coli* O157:H7 is correlated with the subtypes of toxin-encoding phage

Yoshitoshi Ogura,[1] Shakhinur Islam Mondal,[2,3] Md Rakibul Islam,[2] Toshihiro Mako,[4] Kokichi Arisawa,[5] Keisuke Katsura,[2] Tadasuke Ooka,[6] Yasuhiro Gotoh,[2] Kazunori Murase,[2] Makoto Ohnishi,[7] and Tetsuya Hayashi[1]

[1]Department of Bacteriology, Faculty of Medical Sciences, Kyushu University, 3-1-1 Maidashi, Higashi-ku, Fukuoka 812-8582, Japan: [2]Division of Microbiology, Department of Infectious Diseases, Faculty of Medicine, University of Miyazaki, 5200 Kihara, Kiyotake, Miyazaki 889-1692, Japan: [3]Genetic Engineering and Biotechnology Department, Shahjalal University of Science and Technology, Kumargaon, Sylhet-3114, Bangladesh: [4]Environmental Science Division, Fukuoka City Institute for Hygiene and the Environment, 2-1-34 Jigyouhama, Chuou-ku, Fukuoka 810-0065, Japan: [5]Department of Preventive Medicine, Institute of Health Biosciences, University of Tokushima Graduate School, 3-18-15, Kuramoto, Tokushima 770-8504, Japan: [6]Department of Microbiology, Graduate School of Medical and Dental Sciences, Kagoshima University, Sakuragaoka 8-35-1, Kagoshima 890-8544, Japan: [7]Department of Bacteriology, National Institute of Infectious Diseases, 1-23-1 Toyama, Shinjuku-ku, Tokyo 162-8640, Japan.

Email: thayash@bact.med.kyushu-u.ac.jp

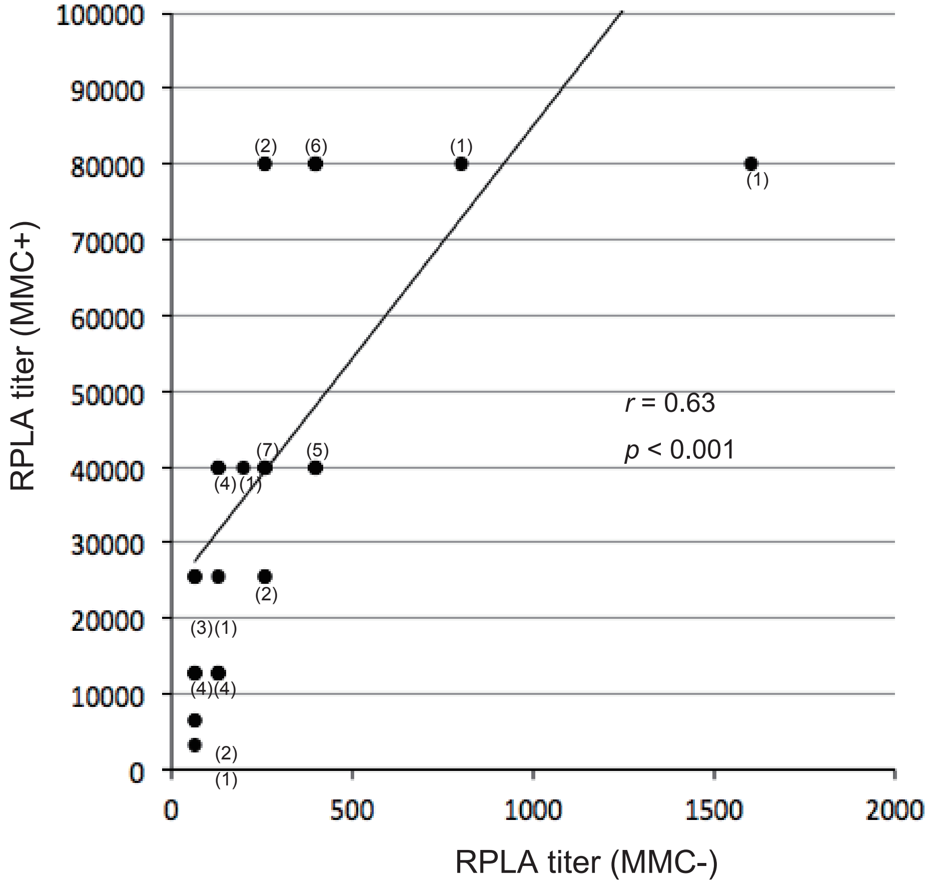

Supplementary Fig. S1. Correlation between the Stx2 production levels in the MMC-treated cells and in the untreated cells.

In each of the 44 strains carrying the *stx2a* gene alone or in combination with the *stx2c* gene, the Stx2 production levels after the MMC treatment were compared to those with no MMC treatment. The Stx2 concentration in each supernatant prepared from the polymixin B-treated culture was measured using the VTEC-Reversed Passive Latex Agglutination (VTEC-RPLA) assay kit. The data (RPLA titers) are the same as those shown in Fig. 1 in the main text. The numbers of strains that yielded the same result (plotted at the same point in the figure) are indicated in parentheses. Pearson' s correlation coefficient (r) and the statistical significance value (p) are indicated.

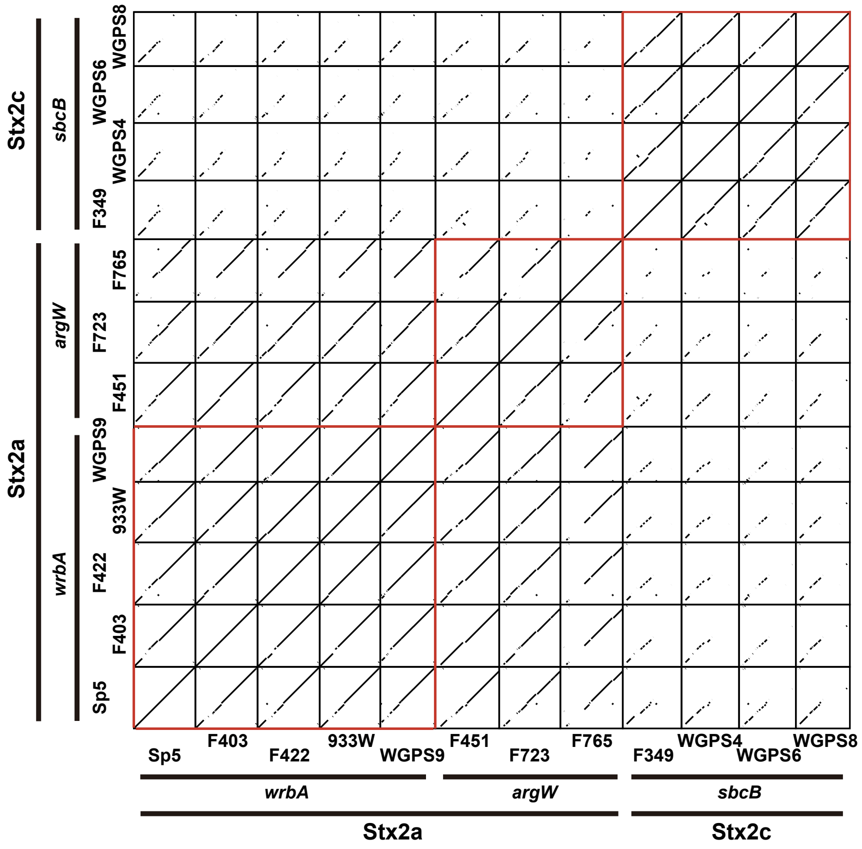

Supplementary Fig. S2. Genome sequence comparison of the Stx2 phages.

A dot-plot matrix of the concatenated genome sequences of the 10 Stx2 phages sequenced in this study and the two Stx2a phages of the previously sequenced O157 strains (Sp5 and 933W of strains Sakai and EDL933, respectively) were generated using the GenomeMatcher software. The Stx2 prophages that were integrated into the same site are indicated with red rectangles.

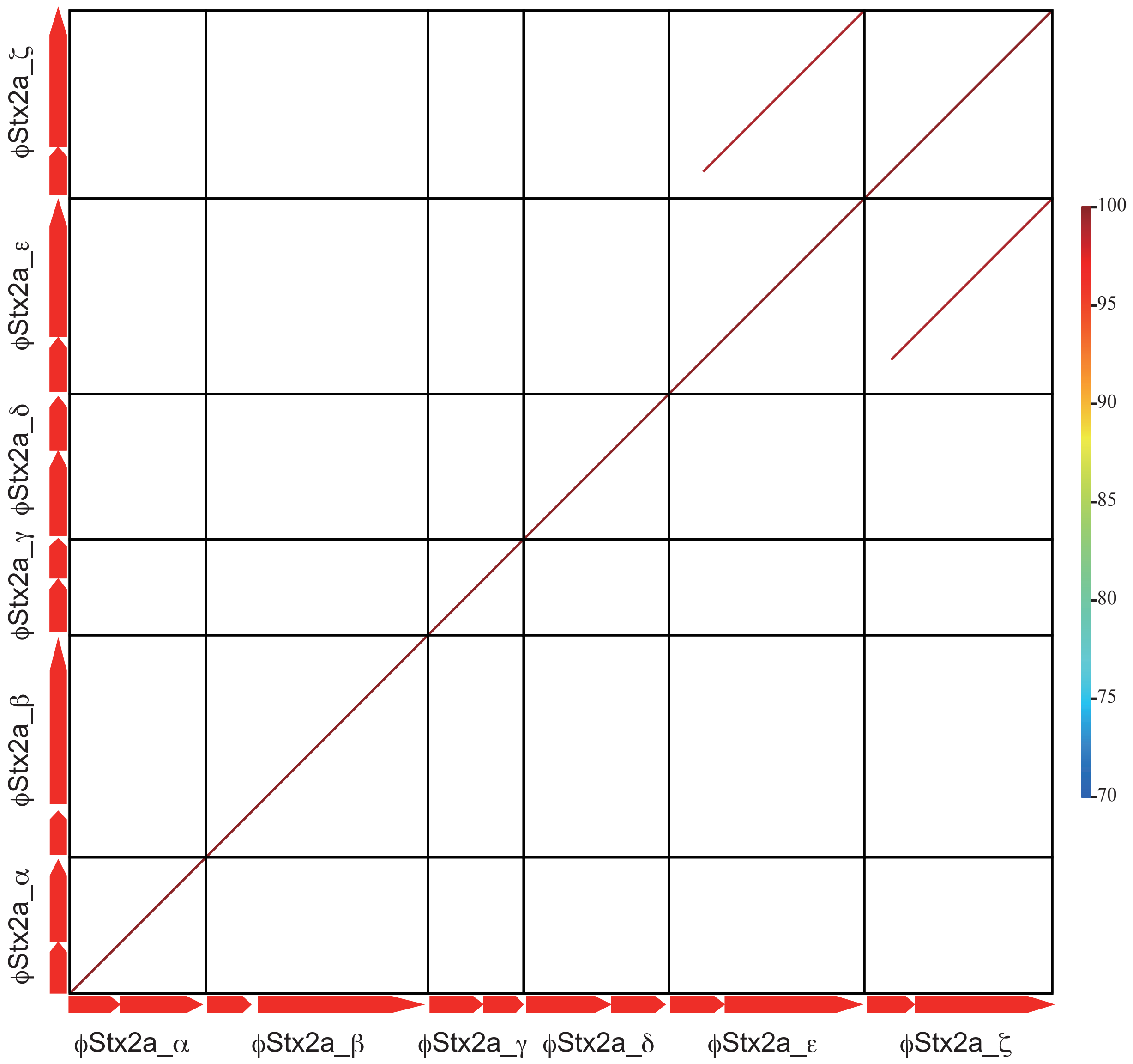

Supplementary Fig. S3. Sequence comparison of the replication genes of each subtype of Stx2a phages. A dot-plot matrix of the concatenated nucleotide sequences of the replication genes of each subtype of Stx2a phages were generated using the GenomeMatcher software. Note that no segment showing >70% nucleotide sequence identity was detected between the replication genes of 6 subtypes except for the segments in phiStx2a_ε and phiStx2a_ζ, which span from the 3'-part of the O gene homolog to the end of the *dnaB* gene homolog.

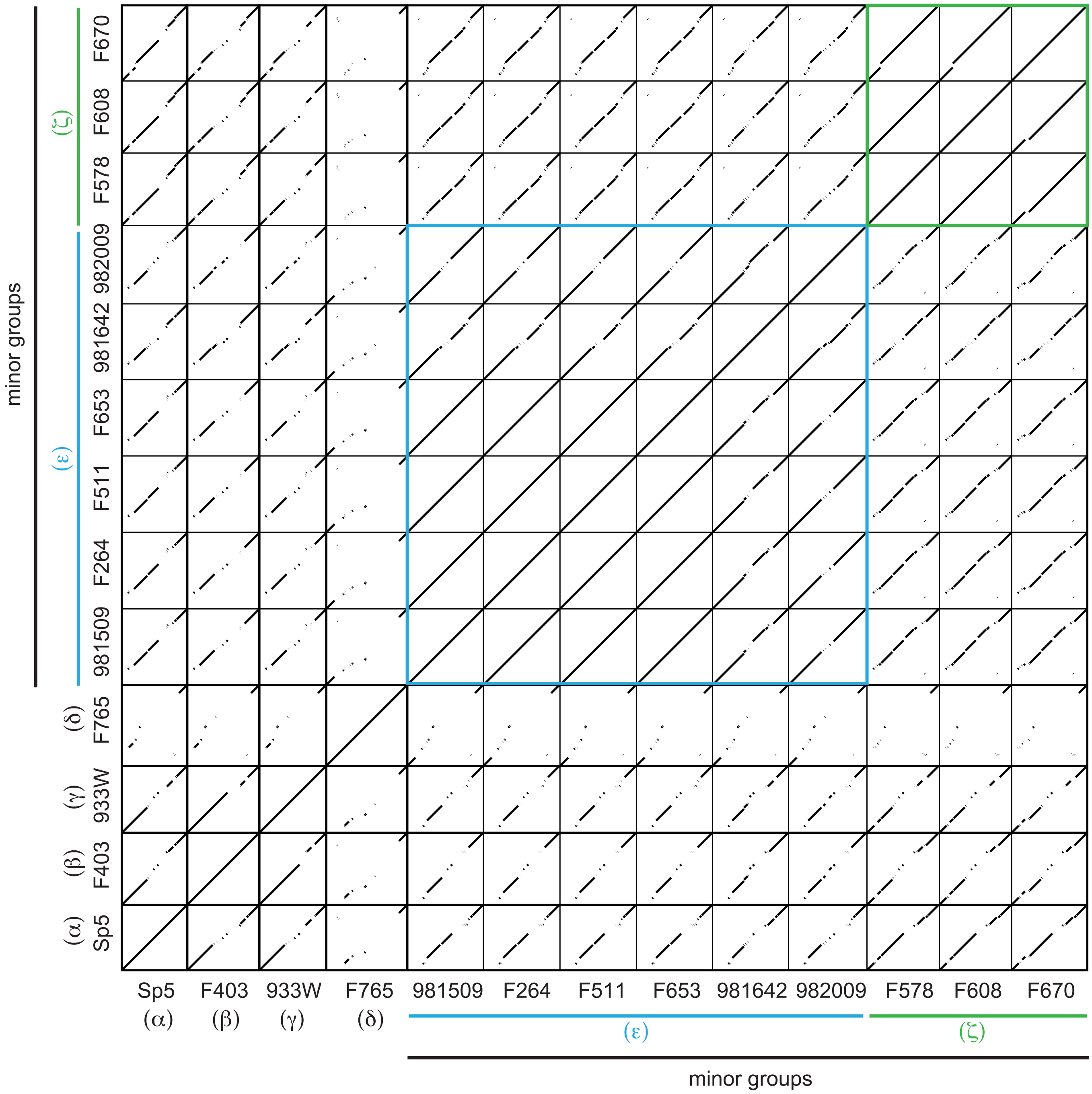

Supplementary Fig. S4. Sequence comparison of the early regions of the four major and two minor subtypes of Stx2a phage.

A dot-plot matrix of the concatenated sequences of early resions of the four major subtypes and two minor subtypes of Stx2a phage were generated using the GenomeMatcher software.

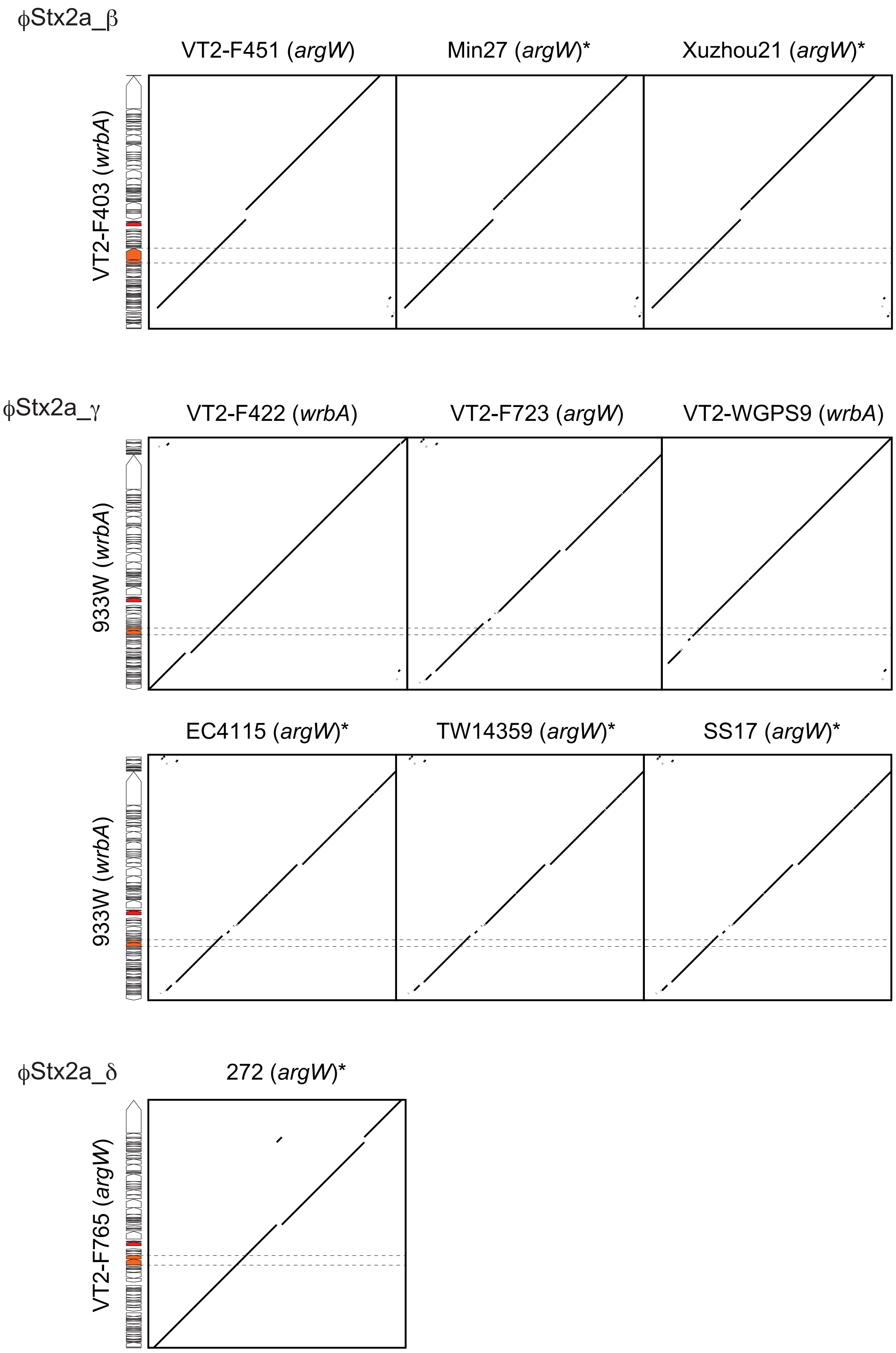

Supplementary Fig. S5. Genome sequence comparison between the Stx2 phages sequenced in this study and Stx2 phages available in public databases.

Dot-plot matrices of the genome sequences of the Stx2 phages analyzed in this study and publically available genome sequences of Stx2 phages from various O157 strains (indicated by asterisks) were generated using the GenomeMatcher software. Genesfor Stx2 and replication proteins are indicated by red and orange, respectively.

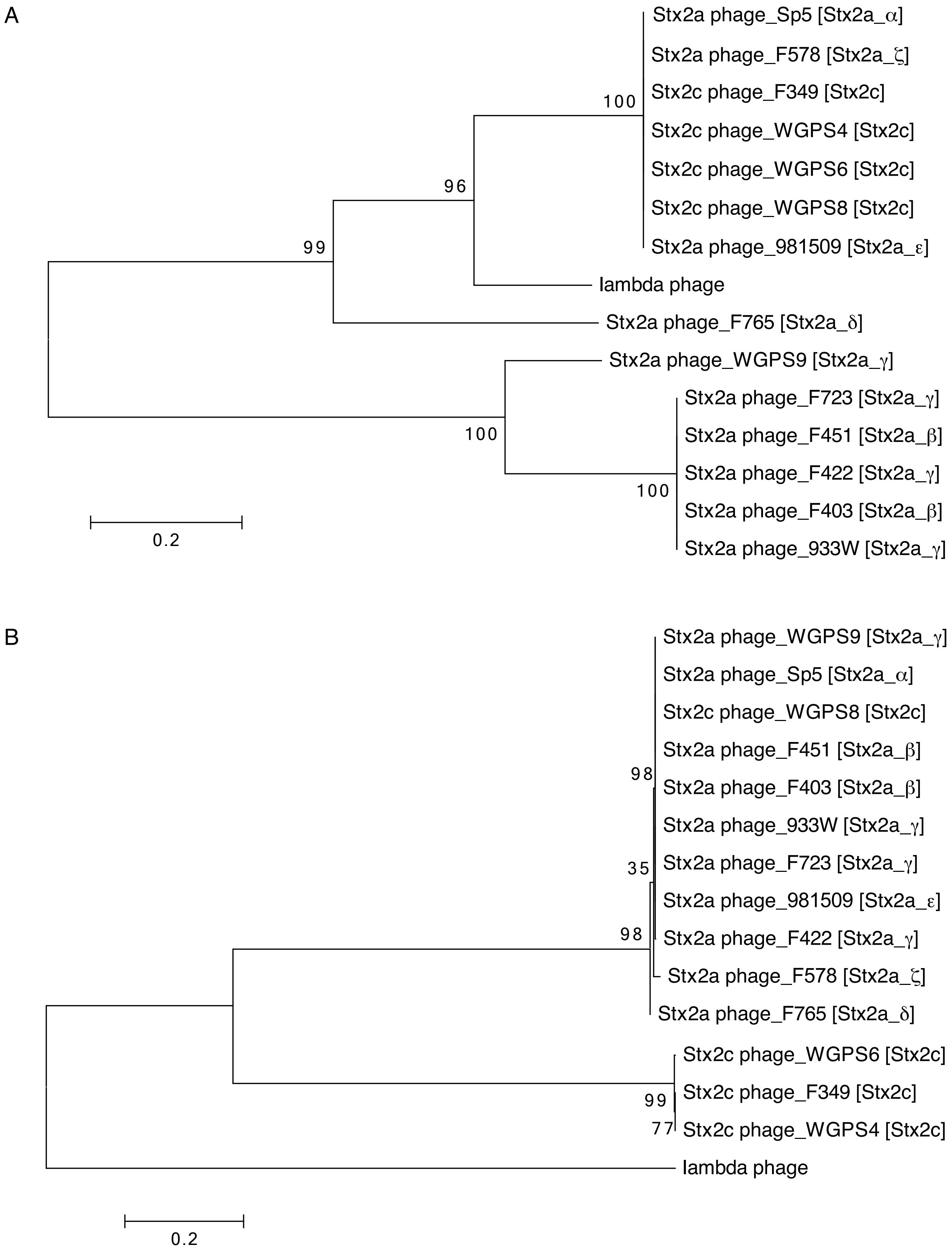

Supplementary Fig. S6. Neighbor-joining (NJ) trees of the *cI* and *q* genes from the Stx2 phages sequenced in this study. Nucleotide sequences of the *cI* and *q* genes (A and B, respectively) of the Stx2a phages sequenced in this study were aligned using the CLUSTALW program. NJ trees were generated using MEGA4. The *cI* and *q* genes of phage lambda and two previously sequenced Stx2 phages (Sp5 and 933W) were included in this analysis. Subtypes of each Stx2 phage are indicated in brackets. The reliabilities of the internal branches were assessed using bootstrapping with 1000 pseudoreplicates. The scale bar represents the number of substitutions per site.

Supplementary Table S1. A list of the O157 strains analyzed in this study and their data summary

strain	Stx2 production (RPLA titer)		toxin type		Stx2 phage integration sites		Stx2a phage subtyping (1st PCR)					Stx2a phage subtyping (2nd PCR)					Stx2a phage subtype	clade	Year of isolation	Symptoms		
	MMC-	MMC+	*stx1*	*stx2* subtype	Stx2a	Stx2c	ϕStx2a_α	ϕStx2a_β	ϕStx2a_γ	ϕStx2a_δ	ϕStx2c	ϕStx2a_α	ϕStx2a_β	ϕStx2a_γ	ϕStx2a_δ	ϕStx2c				diarrhea	bloody diarrhea	HUS
981322	400	40000	+	*stx2a*	*wrbA*	-	+	-	-	-	-	+	nt	nt	nt	nt	ϕStx2a_α	1	1998	+	-	-
982324	400	80000	+	*stx2a*	*wrbA*	-	+	-	-	-	-	+	nt	nt	nt	nt	ϕStx2a_α	1	1998	-	-	-
F691	256	25600	+	*stx2a*	*wrbA*	-	+	-	-	-	-	+	nt	nt	nt	nt	ϕStx2a_α	1	1990s	+	+	-
RIMD 0509952 (Sakai)	256	40000	+	*stx2a*	*wrbA*	-	+	-	-	-	-	+	nt	nt	nt	nt	ϕStx2a_α	1	1996			
981378	64	25600	+	*stx2a*	*wrbA*	-	+	-	-	-	-	+	nt	nt	nt	nt	ϕStx2a_α	2	1998	+	+	-
980551 (WGPS5)	128	40000	+	*stx2a*	*wrbA*	-	+	-	-	-	-	+	nt	nt	nt	nt	ϕStx2a_α	2	1998	+	+	-
F262	256	40000	+	*stx2a*	*wrbA*	-	+	-	-	-	-	+	nt	nt	nt	nt	ϕStx2a_α	2	1990s	+	-	-
F540	400	80000	+	*stx2a*	*wrbA*	-	+	-	-	-	-	+	nt	nt	nt	nt	ϕStx2a_α	2	1990s	+	+	-
F595	128	40000	+	*stx2a*	*wrbA*	-	+	-	-	-	-	+	nt	nt	nt	nt	ϕStx2a_α	2	1990s	+	-	-
F596	256	40000	-	*stx2a*	*wrbA*	-	+	-	-	-	-	+	nt	nt	nt	nt	ϕStx2a_α	2	1990s	+	-	-
F597	200	20000	-	*stx2a*	*wrbA*	-	+	-	-	-	-	+	nt	nt	nt	nt	ϕStx2a_α	2	1990s	+	-	-
F598	200	80000	-	*stx2a*	*wrbA*	-	+	-	-	-	-	+	nt	nt	nt	nt	ϕStx2a_α	2	1990s	+	-	-
F60711	256	40000	+	*stx2a*	*wrbA*	-	+	-	-	-	-	+	nt	nt	nt	nt	ϕStx2a_α	2	2006	+	-	-
F689	256	40000	+	*stx2a*	*wrbA*	-	+	-	-	-	-	+	nt	nt	nt	nt	ϕStx2a_α	2	1990s	+	-	-
F784	256	40000	+	*stx2a*	*wrbA*	-	+	-	-	-	-	+	nt	nt	nt	nt	ϕStx2a_α	2	1990s	+	+	-
F343	128	12800	+	*stx2a*	*wrbA*	-	-	+	-	-	-	nt	+	nt	nt	nt	ϕStx2a_β	3	1990s	+	-	-
F345	128	12800	+	*stx2a*	*wrbA*	-	-	+	-	-	-	nt	+	nt	nt	nt	ϕStx2a_β	3	1990s	+	-	-
F348	64	12800	+	*stx2a*	*wrbA*	-	-	+	-	-	-	nt	+	nt	nt	nt	ϕStx2a_β	3	1990s	+	-	-
F403	64	12800	+	*stx2a*	*wrbA*	-	-	+	-	-	-	nt	+	nt	nt	nt	ϕStx2a_β	3	1990s	+	+	-
F493	200	20000	+	*stx2a*	*wrbA*	-	-	+	-	-	-	nt	+	nt	nt	nt	ϕStx2a_β	3	1990s	+	-	-
F496	128	20000	+	*stx2a*	*wrbA*	-	-	+	-	-	-	nt	+	nt	nt	nt	ϕStx2a_β	3	1990s	+	-	-
F497	200	20000	+	*stx2a*	*wrbA*	-	-	+	-	-	-	nt	+	nt	nt	nt	ϕStx2a_β	3	1990s	+	-	-
F673	32	3200	+	*stx2a*	*wrbA*	-	-	+	-	-	-	nt	+	nt	nt	nt	ϕStx2a_β	3	1990s	+	-	-
F697	64	6400	+	*stx2a*	*wrbA*	-	-	+	-	-	-	nt	+	nt	nt	nt	ϕStx2a_β	3	1990s	+	+	-
F708	64	25600	+	*stx2a*	*wrbA*	-	-	+	-	-	-	nt	+	nt	nt	nt	ϕStx2a_β	3	1990s	+	+	-
F726	128	12800	+	*stx2a*	*wrbA*	-	-	+	-	-	-	nt	+	nt	nt	nt	ϕStx2a_β	3	1990s	+	-	-
F792	128	12800	+	*stx2a*	*wrbA*	-	-	+	-	-	-	nt	+	nt	nt	nt	ϕStx2a_β	3	1990s	+	-	-
F451	64	6400	-	*stx2a*	*argW*	-	-	+	-	-	-	nt	+	nt	nt	nt	ϕStx2a_β	7	1990s	+	-	-
F457	64	6400	-	*stx2a*	*argW*	-	-	+	-	-	-	nt	+	nt	nt	nt	ϕStx2a_β	7	1990s	+	-	-
EDL933	400	40000	+	*stx2a*	*wrbA*	-	-	-	+	-	-	nt	nt	+	nt	nt	ϕStx2a_γ	3	1982			
F422	1600	80000	+	*stx2a*	*wrbA*	-	-	-	+	-	-	nt	nt	+	nt	nt	ϕStx2a_γ	3	1990s	+	-	-
F583	400	80000	+	*stx2a*	*wrbA*	-	-	-	+	-	-	nt	nt	+	nt	nt	ϕStx2a_γ	3	1990s	+	-	-
F70806	256	40000	+	*stx2a*	*wrbA*	-	-	-	+	-	-	nt	nt	+	nt	nt	ϕStx2a_γ	3	2007	+	+	-
F779	400	40000	+	*stx2a*	*wrbA*	-	-	-	+	-	-	nt	nt	+	nt	nt	ϕStx2a_γ	3	1990s	+	+	-
980706 (WGPS3)	400	40000	+	*stx2a+stx2c*	*argW*	*sbcB*	-	-	+	-	+	nt	nt	+	nt	+	ϕStx2a_γ	4/5	1998	+	+	-
990057	400	40000	-	*stx2a+stx2c*	*argW*	*sbcB*	-	-	+	-	+	nt	nt	+	nt	+	ϕStx2a_γ	8	1998	+	+	-
F416	400	80000	-	*stx2a+stx2c*	*argW*	*sbcB*	-	-	+	-	+	nt	nt	+	nt	+	ϕStx2a_γ	8	1990s	-	-	-
F641	256	80000	-	*stx2a*	*argW*	-	-	-	+	-	-	nt	nt	+	nt	nt	ϕStx2a_γ	8	1990s	+	-	-
F723	256	80000	-	*stx2a*	*argW*	-	-	-	+	-	-	nt	nt	+	nt	nt	ϕStx2a_γ	8	1990s	+	+	-
F799	400	40000	-	*stx2a+stx2c*	*argW*	*sbcB*	-	-	+	-	+	nt	nt	+	nt	+	ϕStx2a_γ	8	1990s	+	+	-
F816	400	80000	-	*stx2a+stx2c*	*argW*	*sbcB*	-	-	+	-	+	nt	nt	+	nt	+	ϕStx2a_γ	8	1990s	+	-	-
981795 (WGPS9)	64	25600	+	*stx2a*	*wrbA*	-	-	-	+	-	-	nt	nt	+	nt	nt	ϕStx2a_γ	untypeable	1998	+	+	-
F538	400	80000	+	*stx2a*	*wrbA*	-	-	-	+	-	-	nt	nt	+	nt	nt	ϕStx2a_γ	untypeable	1990s	+	+	+
F550	400	80000	+	*stx2a*	*wrbA*	-	-	-	+	-	-	nt	nt	+	nt	nt	ϕStx2a_γ	untypeable	1990s	+	-	-
F700	800	80000	+	*stx2a*	*wrbA*	-	-	-	+	-	-	nt	nt	+	nt	nt	ϕStx2a_γ	untypeable	1990s	+	-	-
F60604	200	12800	-	*stx2a+stx2c*	*argW*	*sbcB*	-	-	-	+	+	nt	nt	nt	+	+	ϕStx2a_δ	6	2006	+	-	-
F60610	128	12800	-	*stx2a+stx2c*	*argW*	*sbcB*	-	-	-	+	+	nt	nt	nt	+	+	ϕStx2a_δ	6	2006	+	-	-
F60713	400	20000	-	*stx2a+stx2c*	*argW*	*sbcB*	-	-	-	+	+	nt	nt	nt	+	+	ϕStx2a_δ	6	2006	+	+	-
F718	128	12800	-	*stx2a+stx2c*	*argW*	*sbcB*	-	-	-	+	+	nt	nt	nt	+	+	ϕStx2a_δ	6	1990s	+	-	-
982234	64	12800	-	*stx2a*	*argW*	-	-	-	-	+	-	nt	nt	nt	+	nt	ϕStx2a_δ	8	1998	-	-	-
982342	200	40000	-	*stx2a*	*argW*	-	-	-	-	+	-	nt	nt	nt	+	nt	ϕStx2a_δ	8	1998	-	-	-
F646	128	40000	-	*stx2a*	*argW*	-	-	-	-	+	-	nt	nt	nt	+	nt	ϕStx2a_δ	8	1990s	+	-	-
F681	128	25600	-	*stx2a*	*argW*	-	-	-	-	+	-	nt	nt	nt	+	nt	ϕStx2a_δ	8	1990s	+	-	-
F690	128	40000	-	*stx2a*	*argW*	-	-	-	-	+	-	nt	nt	nt	+	nt	ϕStx2a_δ	8	1990s	+	-	-
F765	64	12800	-	*stx2a*	*argW*	-	-	-	-	+	-	nt	nt	nt	+	nt	ϕStx2a_δ	8	1990s	+	+	+
981509	64	3200	-	*stx2a+stx2c*	*argW*	*sbcB*	-	-	-	-	+	nt	nt	nt	nt	+	ϕStx2a_ε	7	1998	+	+	-
981642	128	12800	-	*stx2a+stx2c*	*argW*	*sbcB*	-	-	-	-	+	nt	nt	nt	nt	+	ϕStx2a_ε	7	1998	+	-	-
982009	256	25600	-	*stx2a+stx2c*	*argW*	*sbcB*	-	-	-	-	+	nt	nt	nt	nt	+	ϕStx2a_ε	7	1998	-	-	-
F264	2	64	-	*stx2a+stx2c*	*argW*	*sbcB*	-	-	-	-	+	nt	nt	nt	nt	+	ϕStx2a_ε	7	1990s	+	+	-
F511	32	3200	-	*stx2a+stx2c*	*argW*	*sbcB*	-	-	-	-	+	nt	nt	nt	nt	+	ϕStx2a_ε	7	1990s	-	-	-
F653	64	6400	-	*stx2a+stx2c*	*argW*	*sbcB*	-	-	-	-	+	nt	nt	nt	nt	+	ϕStx2a_ε	7	1990s	+	-	-
F578	200	80000	+	*stx2a*	*wrbA*	-	-	-	-	-	-	nt	nt	nt	nt	nt	ϕStx2a_η	3	1990s	+	+	+
F608	400	40000	-	*stx2a*	*wrbA*	-	-	-	-	-	-	nt	nt	nt	nt	nt	ϕStx2a_η	3	1990s	+	-	-

F670	400	80000	+	*stx2a*	*wrbA*	-	-	-	-	-	-	nt	nt	nt	nt	nt	ϕStx2a_η	3	1990s	+	-	-
F528	nt	nt	-	*stx2c*	-	*sbcB*	-	-	-	-	+	nt	nt	nt	nt	+	ϕStx2c only	4/5	1990s	+	+	-
F280	2	8	-	*stx2c*	-	*sbcB*	-	-	-	-	+	nt	nt	nt	nt	+	ϕStx2c only	6	1990s	+	+	-
F707	nt	nt	-	*stx2c*	-	*sbcB*	-	-	-	-	+	nt	nt	nt	nt	+	ϕStx2c only	6	1990s	+	-	-
990281 (WGPS4)	16	3200	-	*stx2c*	-	*sbcB*	-	-	-	-	+	nt	nt	nt	nt	+	ϕStx2c only	7	1998	-	-	-
990570 (WGPS6)	32	3200	-	*stx2c*	-	*sbcB*	-	-	-	-	+	nt	nt	nt	nt	+	ϕStx2c only	7	1998	+	+	-
981456 (WGPS7)	2	16	+	*stx2c*	-	*sbcB*	-	-	-	-	+	nt	nt	nt	nt	+	ϕStx2c only	7	1998	+	-	-
982243 (WGPS8)	4	800	+	*stx2c*	-	*sbcB*	-	-	-	-	+	nt	nt	nt	nt	+	ϕStx2c only	7	1998	+	-	-
F266	2	2	+	*stx2c*	-	*sbcB*	-	-	-	-	+	nt	nt	nt	nt	+	ϕStx2c only	7	1990s	+	-	-
F282	2	8	-	*stx2c*	-	*sbcB*	-	-	-	-	+	nt	nt	nt	nt	+	ϕStx2c only	7	1990s	+	+	-
F296	2	64	-	*stx2c*	-	*sbcB*	-	-	-	-	+	nt	nt	nt	nt	+	ϕStx2c only	7	1990s	+	-	-
F297	nt	nt	-	*stx2c*	-	*sbcB*	-	-	-	-	+	nt	nt	nt	nt	+	ϕStx2c only	7	1990s	+	-	-
F349	2	2	+	*stx2c*	-	*sbcB*	-	-	-	-	+	nt	nt	nt	nt	+	ϕStx2c only	7	1990s	+	-	-
F351	4	400	-	*stx2c*	-	*sbcB*	-	-	-	-	+	nt	nt	nt	nt	+	ϕStx2c only	7	1990s	+	-	-
F409	16	1600	-	*stx2c*	-	*sbcB*	-	-	-	-	+	nt	nt	nt	nt	+	ϕStx2c only	7	1990s	+	+	-
F467	16	1600	-	*stx2c*	-	*sbcB*	-	-	-	-	+	nt	nt	nt	nt	+	ϕStx2c only	7	1990s	+	+	-
F468	nt	nt	-	*stx2c*	-	*sbcB*	-	-	-	-	+	nt	nt	nt	nt	+	ϕStx2c only	7	1990s	+	-	-
F469	nt	nt	-	*stx2c*	-	*sbcB*	-	-	-	-	+	nt	nt	nt	nt	+	ϕStx2c only	7	1990s	+	-	-
F470	nt	nt	-	*stx2c*	-	*sbcB*	-	-	-	-	+	nt	nt	nt	nt	+	ϕStx2c only	7	1990s	-	-	-
F472	nt	nt	-	*stx2c*	-	*sbcB*	-	-	-	-	+	nt	nt	nt	nt	+	ϕStx2c only	7	1990s	-	-	-
F473	nt	nt	-	*stx2c*	-	*sbcB*	-	-	-	-	+	nt	nt	nt	nt	+	ϕStx2c only	7	1990s	-	-	-
F475	nt	nt	-	*stx2c*	-	*sbcB*	-	-	-	-	+	nt	nt	nt	nt	+	ϕStx2c only	7	1990s	-	-	-
F476	nt	nt	-	*stx2c*	-	*sbcB*	-	-	-	-	+	nt	nt	nt	nt	+	ϕStx2c only	7	1990s	+	-	-
F477	nt	nt	-	*stx2c*	-	*sbcB*	-	-	-	-	+	nt	nt	nt	nt	+	ϕStx2c only	7	1990s	+	-	-
F479	nt	nt	-	*stx2c*	-	*sbcB*	-	-	-	-	+	nt	nt	nt	nt	+	ϕStx2c only	7	1990s	-	-	-
F491	nt	nt	-	*stx2c*	-	*sbcB*	-	-	-	-	+	nt	nt	nt	nt	+	ϕStx2c only	7	1990s	-	-	-
F501	2	16	+	*stx2c*	-	*sbcB*	-	-	-	-	+	nt	nt	nt	nt	+	ϕStx2c only	7	1990s	+	-	-
F517	2	64	+	*stx2c*	-	*sbcB*	-	-	-	-	+	nt	nt	nt	nt	+	ϕStx2c only	7	1990s	+	+	-
F543	nt	nt	-	*stx2c*	-	*sbcB*	-	-	-	-	+	nt	nt	nt	nt	+	ϕStx2c only	7	1990s	+	-	-
F554	nt	nt	-	*stx2c*	-	*sbcB*	-	-	-	-	+	nt	nt	nt	nt	+	ϕStx2c only	7	1990s	+	-	-
F567	16	800	+	*stx2c*	-	*sbcB*	-	-	-	-	+	nt	nt	nt	nt	+	ϕStx2c only	7	1990s	+	-	-
F568	nt	nt	-	*stx2c*	-	*sbcB*	-	-	-	-	+	nt	nt	nt	nt	+	ϕStx2c only	7	1990s	+	-	-
F575	nt	nt	+	*stx2c*	-	*sbcB*	-	-	-	-	+	nt	nt	nt	nt	+	ϕStx2c only	7	1990s	+	-	-
F588	nt	nt	-	*stx2c*	-	*sbcB*	-	-	-	-	+	nt	nt	nt	nt	+	ϕStx2c only	7	1990s	+	+	-
F589	nt	nt	-	*stx2c*	-	*sbcB*	-	-	-	-	+	nt	nt	nt	nt	+	ϕStx2c only	7	1990s	+	-	-
F609	nt	nt	-	*stx2c*	-	*sbcB*	-	-	-	-	+	nt	nt	nt	nt	+	ϕStx2c only	7	1990s	+	-	-
F611	nt	nt	-	*stx2c*	-	*sbcB*	-	-	-	-	+	nt	nt	nt	nt	+	ϕStx2c only	7	1990s	+	-	-
F612	nt	nt	-	*stx2c*	-	*sbcB*	-	-	-	-	+	nt	nt	nt	nt	+	ϕStx2c only	7	1990s	+	+	-
F654	nt	nt	+	*stx2c*	-	*sbcB*	-	-	-	-	+	nt	nt	nt	nt	+	ϕStx2c only	7	1990s	+	+	-
F675	nt	nt	-	*stx2c*	-	*sbcB*	-	-	-	-	+	nt	nt	nt	nt	+	ϕStx2c only	7	1990s	+	-	-
F680	nt	nt	-	*stx2c*	-	*sbcB*	-	-	-	-	+	nt	nt	nt	nt	+	ϕStx2c only	7	1990s	+	+	-
F727	8	256	+	*stx2c*	-	*sbcB*	-	-	-	-	+	nt	nt	nt	nt	+	ϕStx2c only	7	1990s	+	-	-
F786	2	2	+	*stx2c*	-	*sbcB*	-	-	-	-	+	nt	nt	nt	nt	+	ϕStx2c only	7	1990s	+	-	-
F787	2	16	+	*stx2c*	-	*sbcB*	-	-	-	-	+	nt	nt	nt	nt	+	ϕStx2c only	7	1990s	+	-	-
F788	2	16	+	*stx2c*	-	*sbcB*	-	-	-	-	+	nt	nt	nt	nt	+	ϕStx2c only	7	1990s	+	-	-
F796	2	16	+	*stx2c*	-	*sbcB*	-	-	-	-	+	nt	nt	nt	nt	+	ϕStx2c only	7	1990s	+	-	-
F806	2	256	+	*stx2c*	-	*sbcB*	-	-	-	-	+	nt	nt	nt	nt	+	ϕStx2c only	7	1990s	+	-	-
F811	nt	nt	+	*stx2c*	-	*sbcB*	-	-	-	-	+	nt	nt	nt	nt	+	ϕStx2c only	7	1990s	+	-	-
F813	nt	nt	+	*stx2c*	-	*sbcB*	-	-	-	-	+	nt	nt	nt	nt	+	ϕStx2c only	7	1990s	+	-	-
F60607	nt	nt	-	*stx2c*	-	*sbcB*	-	-	-	-	+	nt	nt	nt	nt	+	ϕStx2c only	7	2006	+	-	-
F60608	nt	nt	-	*stx2c*	-	*sbcB*	-	-	-	-	+	nt	nt	nt	nt	+	ϕStx2c only	7	2006	+	-	-
F60611	nt	nt	-	*stx2c*	-	*sbcB*	-	-	-	-	+	nt	nt	nt	nt	+	ϕStx2c only	7	2006	+	-	-
F60616	nt	nt	-	*stx2c*	-	*sbcB*	-	-	-	-	+	nt	nt	nt	nt	+	ϕStx2c only	7	2006	-	-	-
F60617	nt	nt	-	*stx2c*	-	*sbcB*	-	-	-	-	+	nt	nt	nt	nt	+	ϕStx2c only	7	2006	-	-	-
F61007	nt	nt	+	*stx2c*	-	*sbcB*	-	-	-	-	+	nt	nt	nt	nt	+	ϕStx2c only	7	2006	+	-	-
F61008	nt	nt	+	*stx2c*	-	*sbcB*	-	-	-	-	+	nt	nt	nt	nt	+	ϕStx2c only	7	2006	+	-	-
F61009	nt	nt	+	*stx2c*	-	*sbcB*	-	-	-	-	+	nt	nt	nt	nt	+	ϕStx2c only	7	2006	+	-	-
F61010	nt	nt	+	*stx2c*	-	*sbcB*	-	-	-	-	+	nt	nt	nt	nt	+	ϕStx2c only	7	2006	+	-	-
F61021	nt	nt	+	*stx2c*	-	*sbcB*	-	-	-	-	+	nt	nt	nt	nt	+	ϕStx2c only	7	2006	+	+	-
F61025	nt	nt	+	*stx2c*	-	*sbcB*	-	-	-	-	+	nt	nt	nt	nt	+	ϕStx2c only	7	2006	+	-	-

Supplementary Table S2. The list of the primers used in this study

Name	Sequence (5'-3')	Experiments used	Reference
VT1-a	GAAGAGTCCGTGGGATTACG	Detection of the *stx1* gene	Pollard *et. al. J Infect Dis.* **162**, 1195-8 (1990).
VT1-b	AGCGATGCAGCTATTAATAA	Detection of the *stx1* gene	Pollard *et. al. J Infect Dis.* **162**, 1195-8 (1990).
VT2-e	AATACATTATGGGAAAGTAATA	Detection and subtyping of the *stx2* gene	Piérard *et. al. J Clin Microbiol.* **36**, 3317-22 (1998)
VT2-f	TAAACTGCACTTCAGCAAAT	Detection and subtyping of the *stx2* gene	Piérard *et. al. J Clin Microbiol.* **36**, 3317-22 (1998)
stx2-F	GGCGCGTTTTGACCATCTTCGT	Screening of *stx2*-containig fosmid clones	Ohnishi *et. al. Proc Natl Acad Sci U S A.* **99**, 17043-8 (2002)
stx2-R	TACCTTTAGCACAATCCGCCGC	Identification of Stx2 phage integration sites, screening of *stx2* containig fosmid clones, and Stx2a phage subtyping (1st PCR)	Ohnishi *et. al. Proc Natl Acad Sci U S A.* **99**, 17043-8 (2002)
wrbA-R	GGGAATATGTCGCAGGTCTGGC	Identification of Stx2 phage integration sites	this study
ECs3231-R (*argW* in O157)	AAGCAGTACAGCGTTCTCACAG	Identification of Stx2 phage integration sites,	this study
209R (*sbcB*)	GTTGGGCGATCTGGAACAAGTC	Identification of the Stx2 phage integration site	Ohnishi *et. al. Proc Natl Acad Sci U S A.* **99**, 17043-8 (2002)
Stx2a_ST_α	ATGTAATGACTGACGCAGGAG	Stx2a phage subtyping (1st PCR for α subtype)	this study
Stx2a_ST_β	AACATGTCCGAACAGAGTGTG	Stx2a phage subtyping (1st PCR for β subtype)	this study
Stx2a_ST_γ	GCTGATTGAGGCTTATTCAGG	Stx2a phage subtyping (1st PCR for γ subtype)	this study
Stx2a_ST_δ	GTGCATAGGTAGAGCGTTACG	Stx2a phage subtyping (1st PCR for δ subtype)	this study
Stx2a_ST_ε	AAGCGAATTTCAAAGGTCATG	Stx2a phage subtyping (1st PCR for ε subtype)	this study
Stx2a_ST_ζ	CACAAACACGCAGATAGCAGC	Stx2a phage subtyping (1st PCR for ζ subtype)	this study
Stx2a_ST_α_nsF	GTTAGCGACCTTGAGAAGCG	Stx2a phage subtyping (2nd PCR for α subtype)	this study
Stx2a_ST_α_nsR	GTTAACACCTGACTTCTGTCC	Stx2a phage subtyping (2nd PCR for α subtype)	this study
Stx2a_ST_β_nsF	GTGAACGGTCACGGAAAGGC	Stx2a phage subtyping (2nd PCR for β subtype)	this study
Stx2a_ST_β_nsR	CCATTGCTAAGACTACCATCC	Stx2a phage subtyping (2nd PCR for β subtype)	this study
Stx2a_ST_γ_nsF	AACTTTGACCGTGAGCAGATG	Stx2a phage subtyping (2nd PCR for γ subtype)	this study
Stx2a_ST_γ_nsR	AATTCGCGCTGTCATACAGG	Stx2a phage subtyping (2nd PCR for γ subtype)	this study
Stx2a_ST_δ_nsF	TGACGTTGTGTGATGAAATCG	Stx2a phage subtyping (2nd PCR for δ subtype)	this study
Stx2a_ST_δ_nsR	TGACTTTCTCAACGTCATTGC	Stx2a phage subtyping (2nd PCR for δ subtype)	this study
Stx2a_ST_ε_nsF	GGTTGAGCGGATGCTTCAGG	Stx2a phage subtyping (2nd PCR for ε subtype)	this study
Stx2a_ST_ζ_nsF	AATCGGAGTGAACAAGGTGG	Stx2a phage subtyping (2nd PCR for ζ subtype)	this study
Stx2a_ST_ε_ζ_nsR	CTCCTACCTTAACCAGTCGC	Stx2a phage subtyping (2nd PCR for ε and ζ subtypes)	this study

Supplementary Table S3. Genome sequencing statistics of the 12 O157 clade 8 strains that were sequenced in this study

strain	F461	F641	F646	F681	F690	F723	F765	F799	F816	982234	982342	990057
Total number of reads	1,074,998	952,186	1,263,112	921,798	1,033,244	995,472	832,614	952,166	1,067,582	980,608	846,256	1,018,292
Total number of bases	161,249,700	142,827,900	189,466,800	138,269,700	154,986,600	149,320,800	124,892,100	142,824,900	160,137,300	147,091,200	126,938,400	152,743,800
Number of contigs (> 100 bp)	1,150	2,886	3,321	2,778	2,888	2,719	2,690	3,229	3,163	3,026	2,517	2,617
Total contig length	5,307,492	5,305,964	5,274,020	5,274,020	5,274,020	5,254,182	5,306,761	5,268,119	5,245,479	5,293,327	5,286,976	5,235,357
coverage	30	27	36	26	29	28	24	27	31	28	24	29
